# Amelioration of Chemotherapy-Induced Intestinal Mucositis by Orally Administered Probiotics in a Mouse Model

**DOI:** 10.1371/journal.pone.0138746

**Published:** 2015-09-25

**Authors:** Chun-Yan Yeung, Wai-Tao Chan, Chun-Bin Jiang, Mei-Lien Cheng, Chia-Yuan Liu, Szu-Wen Chang, Jen-Shiu Chiang Chiau, Hung-Chang Lee

**Affiliations:** 1 Division of Gastroenterology and Nutrition, Department of Pediatrics, MacKay Memorial Hospital, Taipei, Taiwan; 2 Department of Medicine, MacKay Medical College, New Taipei City, Taiwan; 3 MacKay Junior College of Medicine, Nursing and Management, Taipei, Taiwan; 4 Department of Medical Research, MacKay Memorial Hospital, Taipei, Taiwan; 5 Department of Hepatology and Gastroenterology, MacKay Memorial Hospital, Taipei, Taiwan; 6 Division of Gastroenterology and Nutrition, Department of Pediatrics, Hsinchu MacKay Memorial Hospital, Hsinchu, Taiwan; 7 Department of Pediatrics, Taipei Medical University, Taipei, Taiwan; University of Palermo, ITALY

## Abstract

**Background and Aims:**

Intestinal mucositis is a frequently encountered side effect in oncology patients undergoing chemotherapy. No well-established or up to date therapeutic strategies are available. To study a novel way to alleviate mucositis, we investigate the effects and safety of probiotic supplementation in ameliorating 5-FU-induced intestinal mucositis in a mouse model.

**Methods:**

Seventy-two mice were injected saline or 5-Fluorouracil (5-FU) intraperitoneally daily. Mice were either orally administrated daily saline, probiotic suspension of *Lactobacillus casei* variety *rhamnosus* (*Lcr35*) or *Lactobacillus acidophilus* and *Bifidobacterium bifidum* (*LaBi*). Diarrhea score, pro-inflammatory cytokines serum levels, intestinal villus height and crypt depth and total RNA from tissue were assessed. Samples of blood, liver and spleen tissues were assessed for translocation.

**Results:**

Marked diarrhea developed in the 5-FU groups but was attenuated after oral *Lcr35* and *LaBi* administrations. Diarrhea scores decreased significantly from 2.64 to 1.45 and 0.80, respectively (*P<*0.001). Those mice in 5-FU groups had significantly higher proinflammatory cytokine levels (TNF-α: 234.80 vs. 29.10, *P*<0.001, IL-6: 25.13 vs. 7.43, *P*<0.001, IFN-γ: 22.07 vs. 17.06, *P* = 0.137). A repairing of damage in jejunal villi was observed following probiotics administration. We also found TNF-α, IL-1β and IL-6 mRNA expressions were up-regulated in intestinal mucositis tissues following 5-FU treatment (TNF-α: 4.35 vs. 1.18, IL-1β: 2.29 vs. 1.07, IL-6: 1.49 vs. 1.02) and that probiotics treatment suppressed this up-regulation (*P*<0.05). No bacterial translocation was found in this study.

**Conclusions:**

In conclusion, our results show that oral administration of probiotics *Lcr35* and *LaB*i can ameliorate chemotherapy-induced intestinal mucositis in a mouse model. This suggests probiotics may serve as an alternative therapeutic strategy for the prevention or management of chemotherapy-induced mucositis in the future.

## Introduction

Intestinal mucositis is a frequently encountered side effect in oncology patients undergoing chemotherapy. The anti-metabolite 5-Fluorouracil (5-FU) is one of the most commonly used chemotherapeutic agents in clinical oncology due to its ability to exert its cytotoxic effects through incorporation into RNA and DNA and finally inhibit DNA synthesis and to improve tumor-free status and survival rates.[1] However, studies estimate 50%-80% of patients undergoing 5-FU chemotherapy develop clinical intestinal mucositis.[2] Severe ulceration, inflammation and hemorrhage develop throughout the entire gastrointestinal tract, especially in the small intestine.[3] Destruction of the intestinal mucosa results in reduced food and fluid intake, altered gut motility and pH value, colonic crypt damage, and changed composition of the gut microbiota.[4] Mucositis has a huge clinical and economic impact because it may require chemotherapy interruption and discontinuation of therapy.[5] Mucositis therefore ultimately reduces treatment efficacy and patient survival. Finally it prolongs the time and cost of hospitalization.

At present, current managements of intestinal mucositis remain mostly symptomatic treatment including protective mucosal coatings, topical antimicrobials, cryotherapy, antibiotics, and analgesics.[6] Recent reports have described a decreased severity of intestinal mucositis in murine models investigating agents including insulin-like growth factor-1, keratinocyte growth factor, glucagon-like peptide and epidermal growth factor 1.[3,6–8] However, no well-established or up-to-date therapeutic strategies to manage chemotherapy-induced intestinal mucositis are available. Thus the development of an effective intervention against chemotherapy-related mucositis has high priority in oncological supportive care.

Probiotics are defined as ‘live micro-organisms which, when administered in adequate amounts, confer a health benefit on the host’.[9] Recently, probiotics have been investigated as a therapeutic approach in a range of disorders, such as inflammatory bowel disease, colitis, pouchitis, enteric infection, irritable bowel syndrome, colon cancer, radiation induced enteropathy and chemotherapy-induced mucositis.[10–12] Recently, a dysbiosis theory has been described in 5-FU-induced mucositis which is likely to contribute to the general development of mucositis.[13]

Our previous researches demonstrated that *Lactobacillus* could attenuate the barrier disruption of intestinal epithelial cells caused by *Salmonella* lipopolysaccharide (LPS) administration.[14,15] In light of this finding, we suggest that probiotic may ameliorate inflammation and protect epithelium by maintaining the tight junction integrity and potentially reduce the severity of mucositis. To study a novel way to alleviate mucositis, we investigate the effects of probiotic supplementation in ameliorating 5-FU-induced intestinal mucositis in an experimental mouse model. We also explore the safety of probiotic administration by examining possible translocation of probiotic strains to the blood, liver and spleen.

## Materials and Methods

### 5-FU Treatment

5-FU (Fluorouracil-TEVA^®^, Netherland) was injected intraperitoneally (IP) at a single dose of 30 mg/kg/day for 5 days to cause mucositis and diarrhea as described in the literature.[16] IP saline was injected for alternative in control groups.

### Probiotics Preparation

*Lactobacillus casei* variety *rhamnosus* (*Lcr35*, Antibiophilus^®^, France) and *Lactobacillus acidophilus* and *Bifidobacterium bifidum* (*LaBi*, Infloran^®^, Italy) were used in this experiment. Probiotics were diluted in sterile saline and administered by oral gavages. The mice received 100 μL of saline or suspension containing 1x10^7^ CFU of the probiotics cocktail daily for 5 days.

### Animal Trial

All experiments described were conducted on male Balb/c mice obtained from Taiwan’s National Laboratory Animal Center under a 12h light/dark cycle with a temperature of 22±1°C and a humidity of 55±10%.[16] Animal studies were approved by the Institutional Animal Care and Use Committee (IACUC) of MacKay Memorial Hospital (Taiwan) (IACUC Number: MMH-A-S-102-08). All mice were given ad libitum access to autoclaved food (Laboratory autoclavable rodent diet 5010) and water. The mice were at the age of 6 weeks with weight 22–24gm and randomly divided into six groups (n = 12). The mice were injected saline (three control groups) or 5-FU (three experimental groups) IP daily for 5 days. Mice in each control group and experimental group were orally administrated saline daily, probiotic suspension of *Lcr35* or *LaBi* respectively.

### Diarrhea Assessment

Stool passages of all the mice were recorded daily. Diarrhea severity was assessed by using Bowen’s score system [17] and was classified into four grades according to the stool consistency: 0, normal stool; 1, slightly wet and soft stool indicating mild diarrhea; 2, wet and unformed stool indicating moderate diarrhea; and 3, watery stool indicating severe diarrhea.

### Inflammatory Cytokines Analysis

Blood was collected from the hearts immediately after those mice were sacrificed. Blood samples were centrifuged to yield serum. Serum levels of pro-inflammatory cytokines (TNF-α, IL-1β, IL-6) were assessed by ELISA assay Kit (R&D Systems, Inc., Minneapolis, USA). All assays were performed according to the manufacturer’s instructions.

### Histological Analysis

A 3-cm ring from the proximal area (close to the duodenojejunal flexure) of each harvested jeunum was processed and fixed in 10% buffered neutral formalin for 2 hours, dehydrated in an ascending series of ethanol concentrations, cleared in xylol, and embedded in paraffin wax. Sections of 4-μm thickness were cut and mounted on glass slides then. Sections were routinely stained with haematoxylin and eosin (HE).[18] HE stained goblet cells were expressed as the number of goblet cells per 10 villus-crypt units as described in the literature.[19,20] The image acquisition phase was done with a 20x magnification objective. Specimens were viewed under a TissueFAXS automatic scanning system, captured by a digital camera and analyzed by HistoQuest software (TissueGnostics, Vienna, Austria).[21] Measurements of villus height (VH) and crypt depth (CD) of the small intestine were determined for whole well orientated villi and crypts per small intestinal tissue section per mouse and the values were averaged.

### RNA extraction and PCR

Total RNA from jejunum and colon tissues were isolated using the TRI Reagent^®^ RNA Isolation Reagent (Sigma Co. Ltd, MO, USA) according to the manufacturer’s instructions for animal tissue. Template cDNA was synthesized from RNA using reverse transcription with Oligo (dT) [18] primers (Fermentas, Vilnius, Lithuania). DNA detection and amplification by real-time quantitative PCR (Q-PCR) was performed using an ABI 7500 Sequence Detection System with system software version 1.2.3 (Applied Biosystems, Singapore). Cytokines, including TNF-α, IL-1β and IL-6, were detected by the Maxima SYBR Green/ROX Q-PCR Master Mix (Applied Biosystems, Warrington, UK), with 100 nM of each of the forward and reverse primers and 1 ng DNA per reaction. PCR cycling was performed as follows: 50°C for 2 min, 95°C for 10 min, and 40 cycles of 95°C for 15 s and 60°C for 1 min. Pairs of oligonucleotide primers specific to TNF-α [22], IL-1β [23], and IL-6 [24] housekeeping gene 18sRNA [25] were used. Q-PCR data were analyzed following the 2^-ΔΔCt^ method using 18sRNA as an endogenous control. Thus, the relative quantity of the target transcript is described as fold increase (RQ, relative quantitation) relative to the reference sample and 18sRNA. Duplicate samples were routinely used for the determination of DNA by Q-PCR and mean values were calculated.

### Safety of Probiotics: Translocation and Infections

Samples of blood, liver and spleen tissues were inoculated in MRS broth for 7 days. Then, the samples were homogenized and seeded with a 0.1 ml on MRS agar plate for 2 days. The bacterial colonies were calculated for translocation assay.[26]

### Statistical Analysis

All parametric data were expressed as the mean ± SE. The statistical significance of differences was analyzed using one-way ANOVA. Data were analyzed with IBM SPSS software (version 21.0; SPSS Institute, Chicago, USA). The results were considered statistically significant at *P*<0.05.

## Results

### Body Weight Change

**Fig 1:**
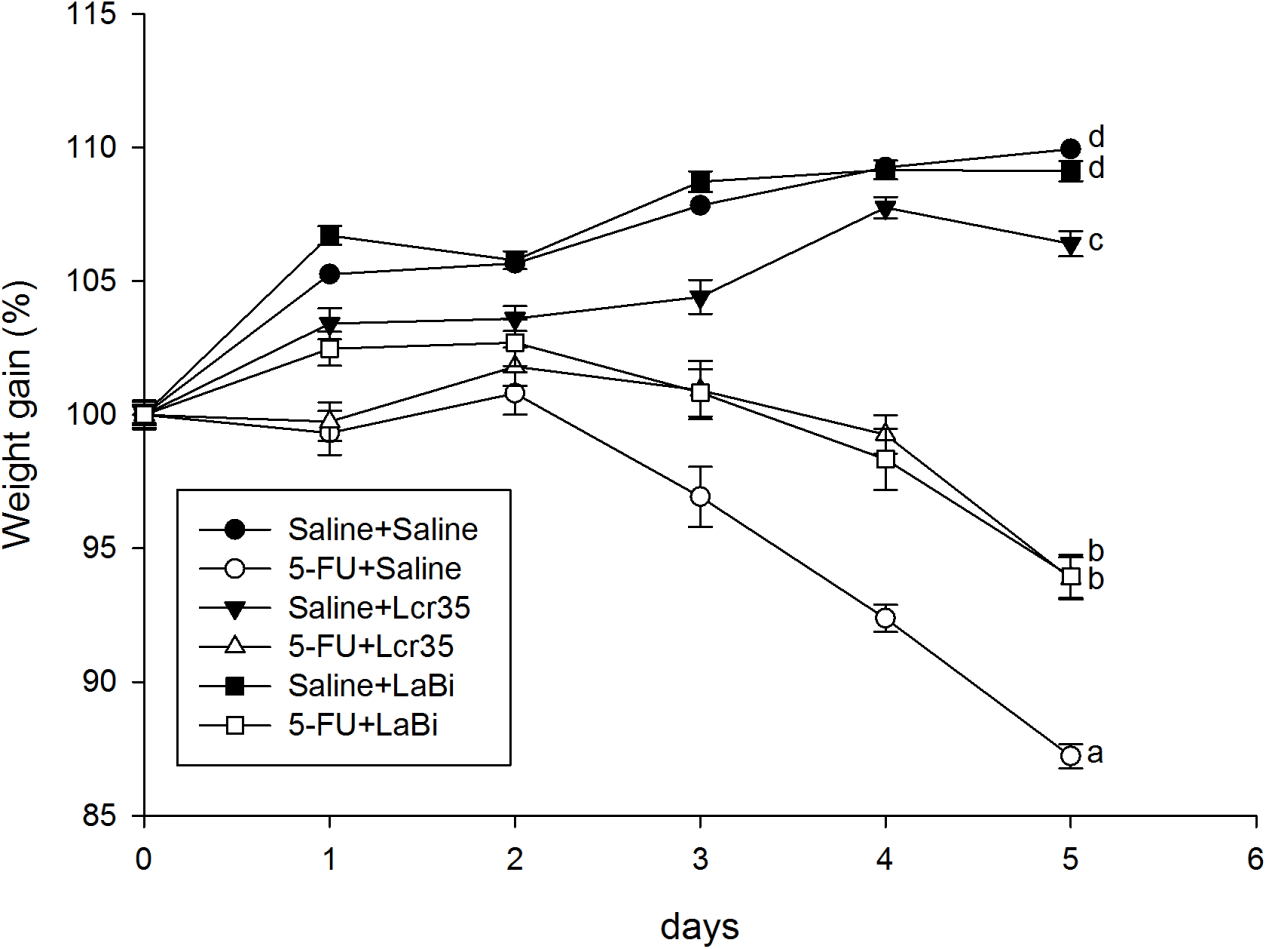
Daily body weight change in percentage of saline or 5-FU-injected mice with/without probiotics (*Lcr35* or *LaBi*) administration. The mice were weighted daily and the results of all groups were compared with those in 5-FU-saline groups for 5 days. In the control groups, the mice were injected saline and administrated with saline (○), *Lcr35* (□) and *LaBi* (△). In the experimental groups, the mice were injected 5-FU and administrated with saline (●), *Lcr35* (■) and *LaBi* (▲). Data of starting bodyweight are expressed 100% from day 0.

After completion of the experiment, all animals tolerate well and no animal exhibited signs of marked adverse effects such as bloody stool passage or cachexia. No mortality was noted. The mice were weighted daily and the results of all groups were compared. Those mice in 5-FU groups had higher body weight (BW) loss than those in saline groups. However, the BW of the mice in 5-FU and *Lcr35* group and 5-FU and *LaBi* group were significantly less (approximately 20%) than those in the 5-FU and saline group (*P* = 0.001) after 5 days (Fig 1).

Following saline administration, all mice gained BW on Day 1, including those treated with *Lcr35* and *LaBi* (P<0.05). A temporal phenomenon was observed following 5-FU injection. We found that BW gain percentage fluctuated initially from Day 1 to Day 2 (99.33±0.83 to 100.80±0.79gm) and then began to decrease from Day 3 to Day 5 (96.93±1.13 to 87.22±0.48gm). Furthermore, in 5-FU injected mice, the decrease in BW was significantly less severe following *Lcr35* and *LaBi* administrations comparing to those without probiotics administration (5-FU+saline group, *P*<0.001). There was no difference between BW loss in 5-FU injected mice treated with either *Lcr35* or *LaBi* (Fig 1).

### Diarrhea Assessment

**Fig 2:**
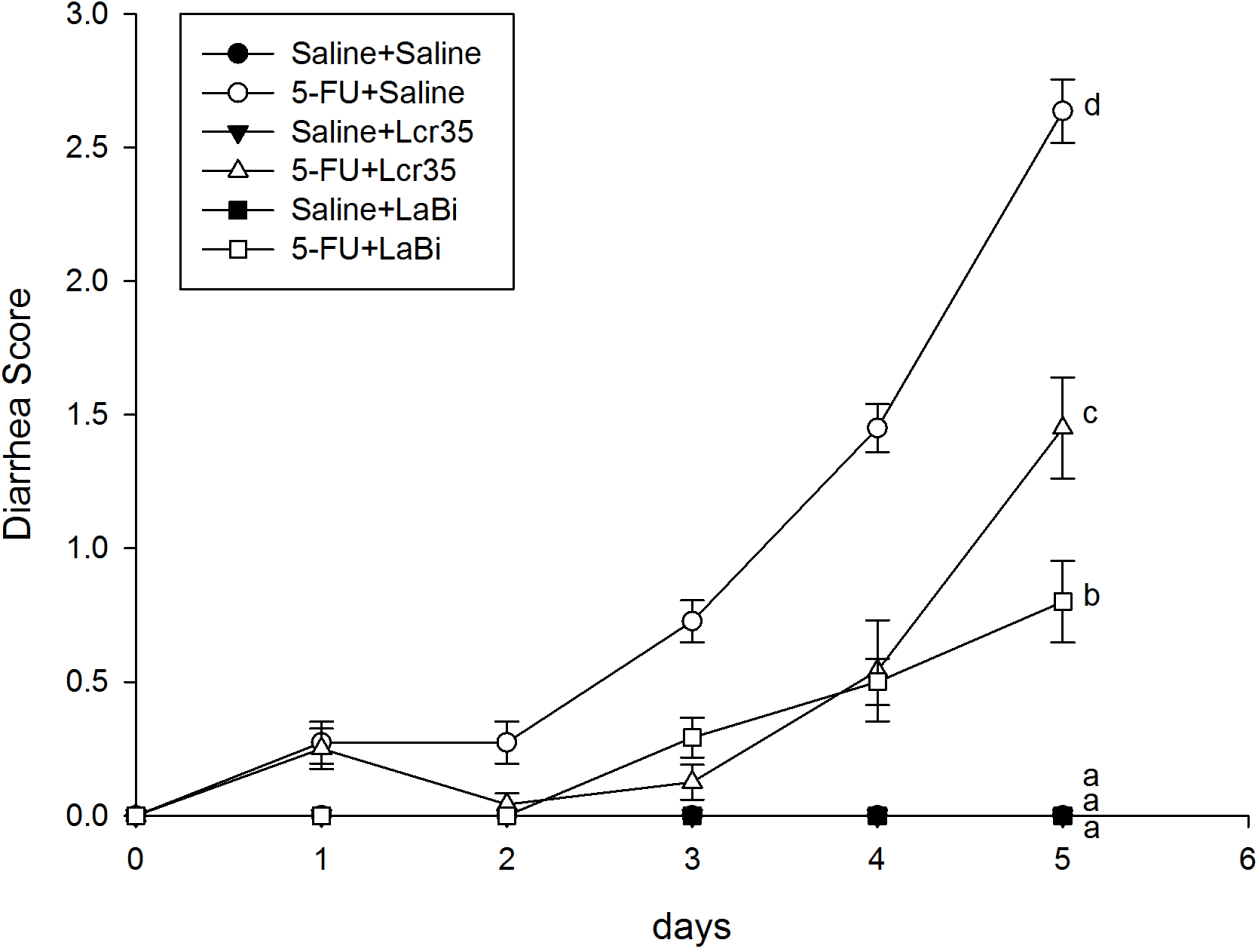
Diarrhea score after administrating probiotics (*Lcr35* or *LaBi*) with/without 5-FU treatment. The mice were recorded daily and the results of all groups were compared with those in 5-FU + saline group for 5 days. In the control groups, the mice injected saline and administrated with saline (○), *Lcr35* (□) and *LaBi* (△). In the experimental groups, the mice injected 5-FU and administrated with saline (●), *Lcr* (■) and *LaBi* (▲). The severity of diarrhea was attenuated in those mice treated with probiotics in the 5-FU groups. The data with different superscripted letters are significantly different based on the one-way ANOVA.

Diarrhea score of the mice were recorded daily and the results of all groups were compared. In the 3 saline groups (with or without probiotics), there were no diarrhea noted. However, marked diarrhea developed in the three 5-FU groups 48 hours later. Diarrhea was attenuated and diarrhea score significantly improved after *Lcr35* and *LaBi* administrations (Fig 2). The severity of diarrhea was clearly attenuated in those mice treated with *Lcr35* and *LaBi* in the 5-FU groups.

### Inflammatory Cytokines Analysis

**Fig 3:**
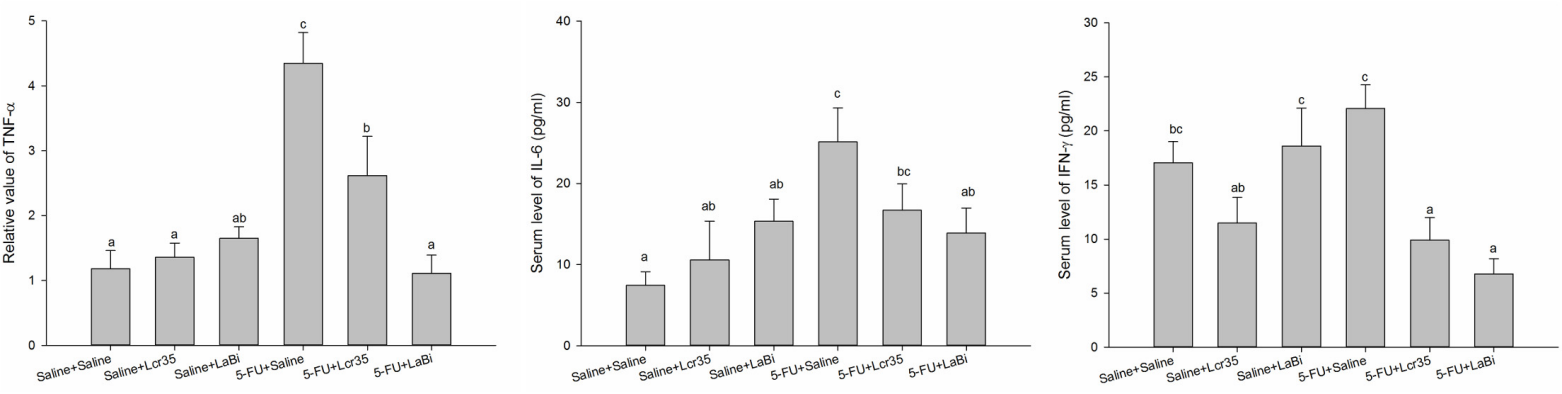
Serum levels of TNF-α, IL-6 and IFN-γ by ELISA assays from mice challenged by 5-FU-induced intestinal mucositis on the 5^th^ day. They were fed with (+) or without (−) probiotics (*Lcr35/LaBi*). The data with different superscripted letters are significantly different based on the one-way ANOVA.

After sacrificed, serum levels of cytokines TNF-α, IL-6 and IFN-γ were assayed and shown in Fig 3. Those mice in 5-FU+saline group had significantly higher circulating proinflammatory cytokine levels than those in saline groups did (TNF-α, 234.80±40.31 vs 29.10±6.69, *P*<0.001; IL-6, 25.13±4.18 vs 7.43±1.68, *P*<0.001; IFN-γ, 22.07±2.20 vs 17.06±1.95, *P* = 0.137). However, for the mice in 5-FU+*Lcr35* and 5-FU+*LaBi* groups, the levels decreased significantly comparing to those mice in the group 5-FU without probiotics administration (5-FU+saline).

### Histological Analysis: Villus height, crypt depth and goblet cells measurements

**Fig 4:**
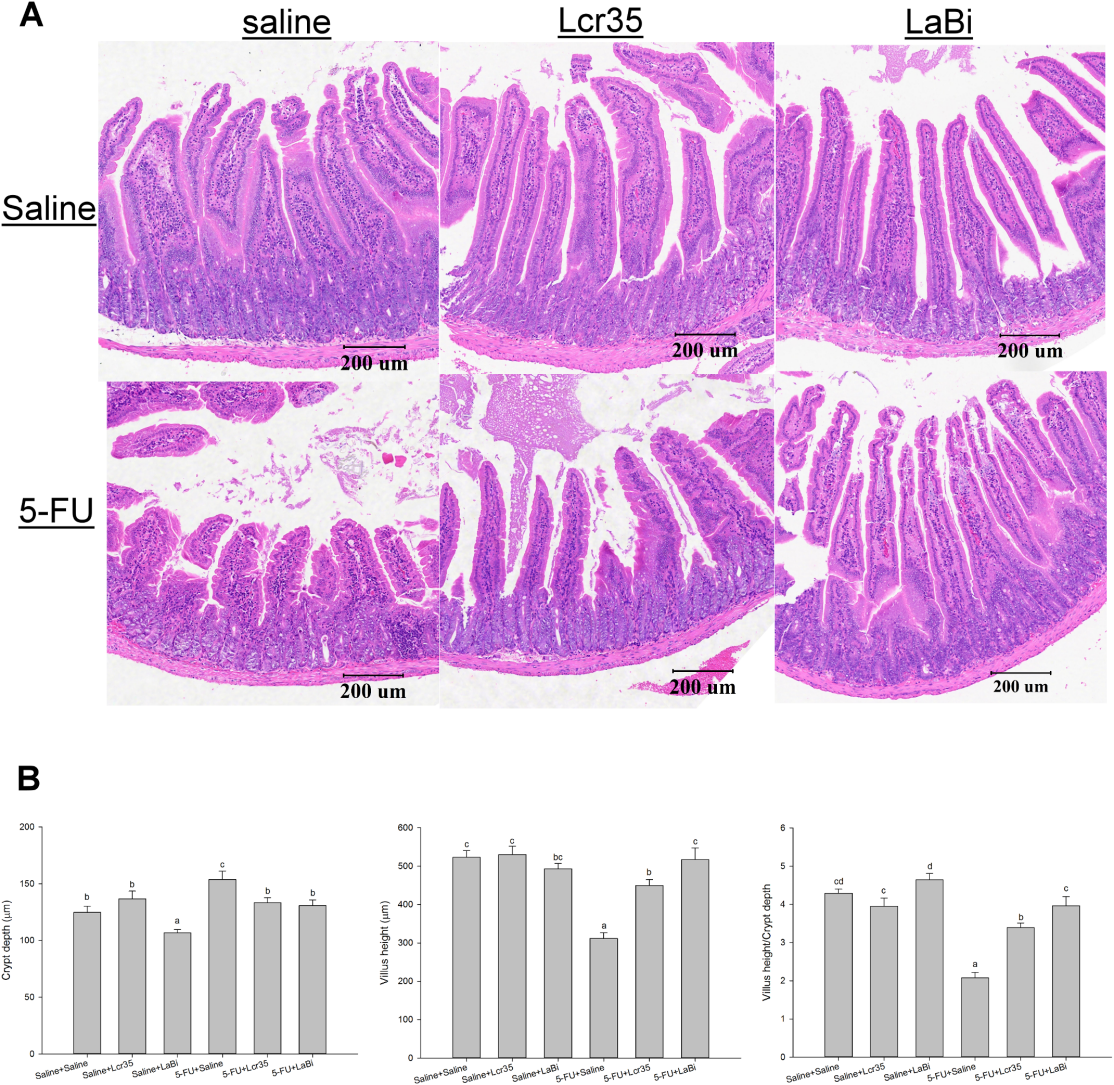
A: Representative histology of jejunum showing villus height and crypt depth with haematoxylin and eosin stain in mice on day 5 challenged with 5-FU (IP). They were fed with probiotics (*Lcr35* or *LaBi*) or saline. The image acquisition phase was done with a 20x magnification objective. Scale bar = 200μm. B: Values were represented as mean ± SEM and were analyzed using one-way ANOVA. Segments of jejunum were taken for measurement of villus height, crypt depth and villus/crypt ratio per mouse.

We examined the probiotics effects on the villus height in the jejunum. 5-FU caused substantial changes in the intestinal mucosal layer (Fig 4A) including flattened epithelial layer, shortened villi and lamina propria with inflammatory cells infiltration. The crypts looked small and narrow. No mitoses were found.

An increased jejunal villus length was observed, however no significant difference was found among the 3 IP saline groups with or without probiotics administration. On the other hand, 5-FU significantly decreased villus height compared to the saline controls (Fig 4B). This effect was restored by *Lcr35* and *LaBi* in 5-FU-injected mice, resulting a significant lengthened jejunal villi compared with 5-FU controls (Fig 4B). Interestingly, we noticed that 5-FU-injected mice treated with *LaBi* resulted in stronger effect compared with *Lcr35* administration. Besides, 5-FU significantly lengthened crypt depth of the intestine compared with the saline controls (Fig 4B). On the contrary, the crypts depth was significantly restored by both *Lcr35* and *LaBi* treatments in 5-FU mice to the levels seen in those normal saline controls. Changes in villus height to crypt depth ratio was similar to that of villus height. 5-FU markedly decreased the ratio in jejunal sections compared to the saline controls (2.07 ± 0.14 vs 4.29 ± 0.12, *P*<0.001), However, these effects were normalized by *Lcr35* (3.39 ± 0.12, *P*<0.001) and *LaB*i (3.96 ± 0.54, *P*<0.001) administrations in 5-FU-injected mice.

**Fig 5:**
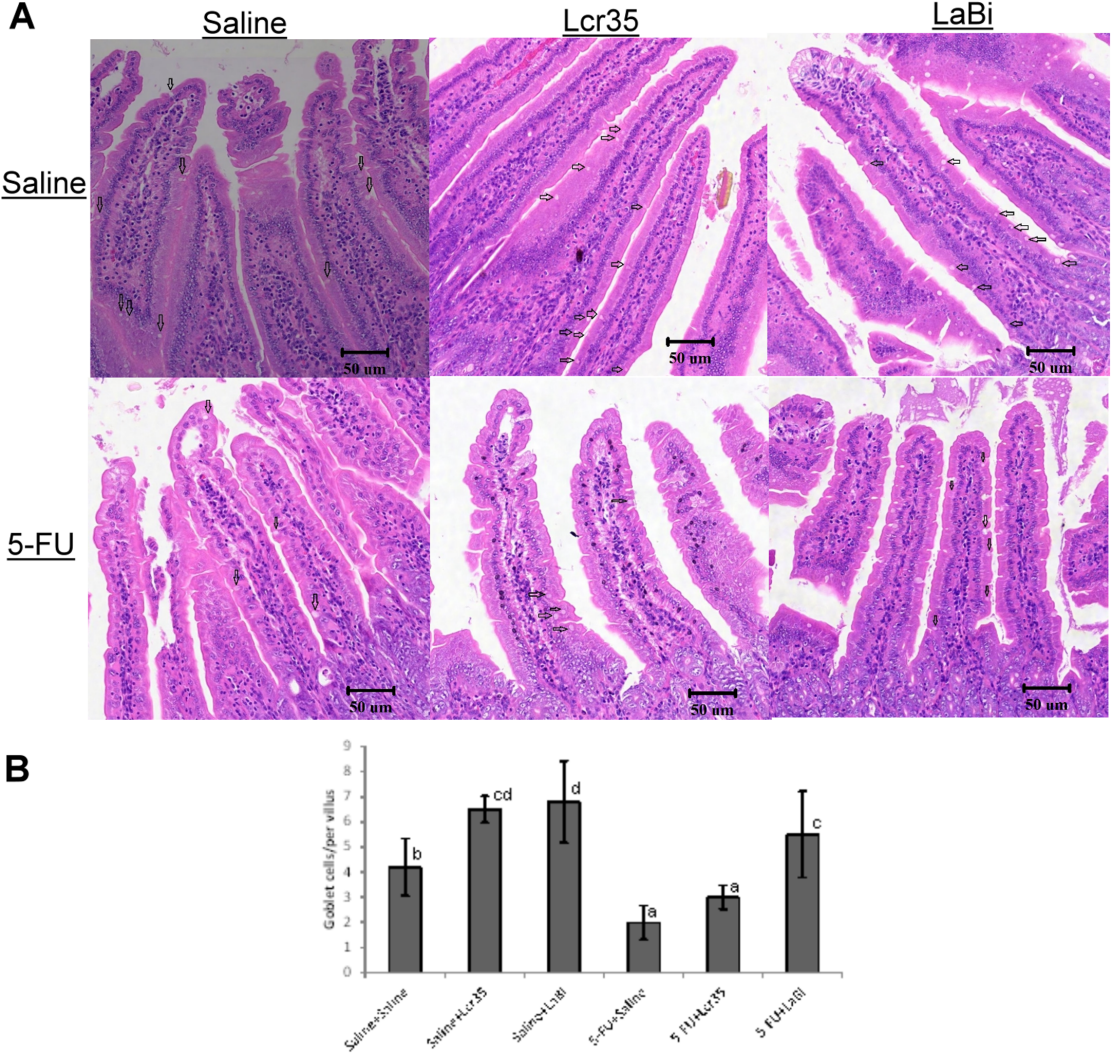
Up-regulations of IL-6, IL-1β and TNF-α in mucositis mice were followed after injection with 5-FU. Mucositis mice were fed with (+) or without (−) probiotics. Gene expressions of IL-6, IL-1β and TNF-α were determined by Q-PCR (A) jejunum tissue (B) colon tissue. Induction of cytokine expressions were presented as RQ compared to 18sRNA housekeeping gene expression. The data with different superscripted letters are significantly different based on the one-way ANOVA.

Besides, goblet cells in the jejunum was counted per villus/crypt in the jejunum. Similar to previous findings in villus height, an increasing number was observed among the 3 IP saline groups with or without probiotics administration (Fig 5A). However, the jejunum exhibited a significant decrease in total goblet cell numbers after treatment with 5-FU (4.2±1.1 vs 2.0±0.7, P<0.05, Fig 5B). This effect was alleviated by *Lcr35* and *LaBi* in 5-FU-injected mice, resulting a significant increase of goblet cell numbers compared with 5-FU controls (Fig 5B). We also noticed that 5-FU-injected mice treated with *LaBi* resulted in significant effect compared with *Lcr35* administration (5.5±1.7 vs 3.0±0.5, p<0.01).

### mRNA assay

**Fig 6:**
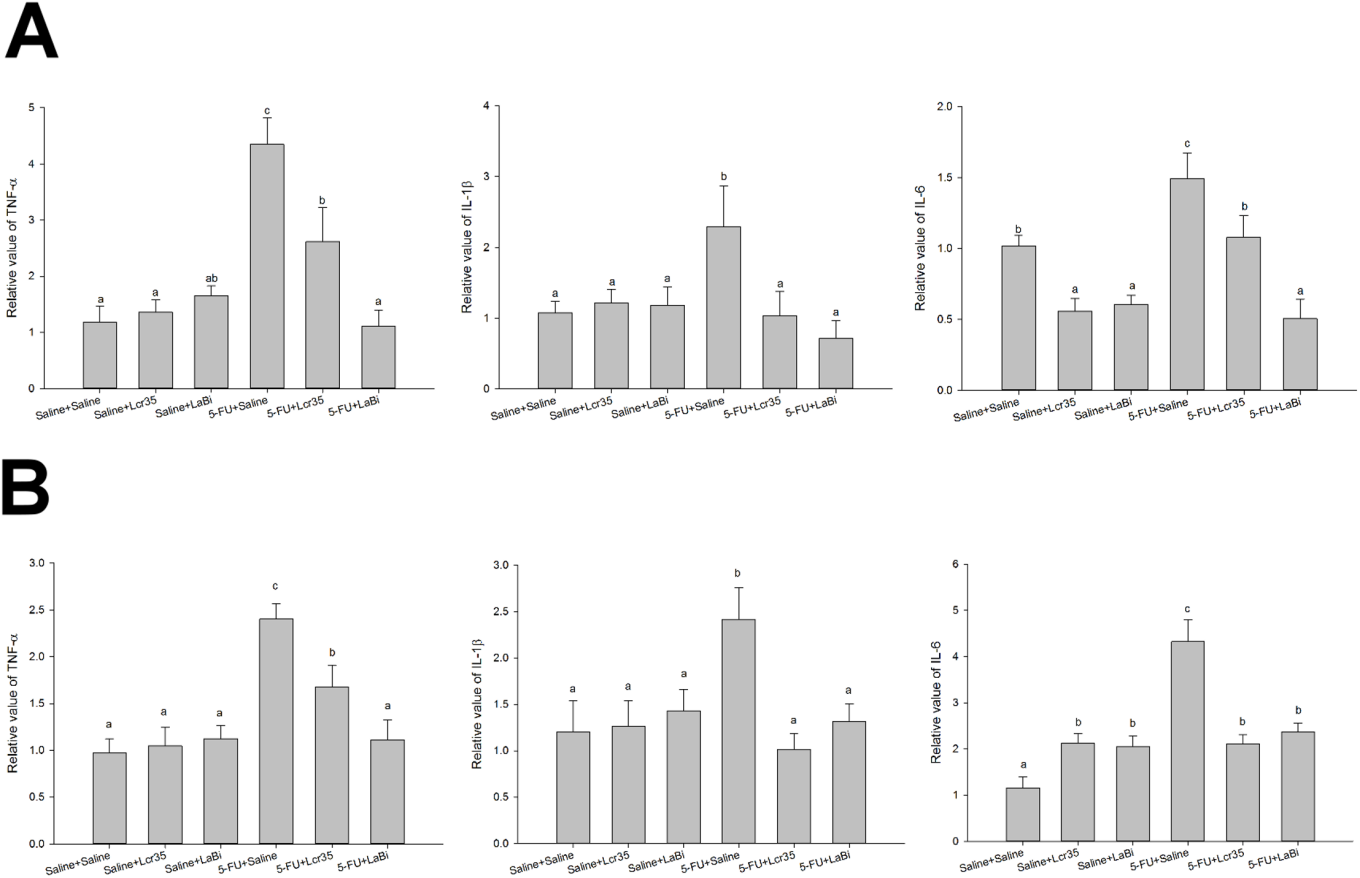
A: Representative histological sections of jejunum showing the goblet cells with haematoxylin and eosin stain in mice on day 5 challenged with 5-FU (IP). They were fed with probiotics (*Lcr35* or *LaBi*) or saline. The arrows indicated goblet cells. The image acquisition phase was done with a 20x magnification objective. Scale bar = 50μm. B: Jejunal goblet cells after staining were counted. Values were represented as mean ± SEM and were analyzed using one-way ANOVA.

Effects of probiotics treatment on TNF-α, IL-1β and IL-6 mRNA expressions in jejunum and colon tissues treated with 5-FU were determined. We found that these expressions were markedly up-regulated and that probiotics treatment suppressed this up-regulations in jejunum tissues (*Lcr35*: TNF-α, 2.62 ± 0.61; IL-1β, 1.04 ± 0.34; IL-6, 1.08 ± 0.18 and *LaBi*: TNF-α, 1.11 ± 0.28; I IL-1β, 0.71±0.26; IL-6, 0.51±0.14) (*P*<0.05) (Fig 6A). We also noticed similar TNF-α, and IL-1β mRNA expressions in colon tissues (Fig 6B).

### Safety and Translocation

Concerning the safety of probiotics administrations, we checked the samples of blood, liver and spleen tissues and calculated the bacterial colonies for translocation assay. No bacterial translocation was found in these samples (Table 1).

**Table 1:** Translocation of probiotics to blood, liver and spleen of 5-FU treated mice fed with (+) or without (−) probiotics on the 5^th^ day was assessed. The bacteria were detected by using Q-PCR (n = 11–13 per group).

	Blood			Liver			Spleen		
	*Lcr35*	*LaBi*		*Lcr35*	*LaBi*		*Lcr35*	*LaBi*	
		*Lactobacillus*	*Bifidobacterium*		*Lactobacillus*	*Bifidobacterium*		*Lactobacillus*	*Bifidobacterium*
**Saline+Saline**	0/12	0/12	0/12	0/12	0/12	0/12	0/12	0/12	0/12
**Saline+*Lcr35***	0/12	0/12	0/12	0/12	0/12	0/12	0/12	0/12	0/12
**Saline+*LaBi***	0/13	0/13	0/13	0/13	0/13	0/13	0/13	0/13	0/13
**5-FU+Saline**	0/11	0/11	0/11	0/11	0/11	0/11	0/11	0/11	0/11
**5-FU+*Lcr35***	0/12	0/12	0/12	0/12	0/12	0/12	0/12	0/12	0/12
**5-FU+*LaBi***	0/12	0/12	0/12	0/12	0/12	0/12	0/12	0/12	0/12

## Discussion

Intestinal mucositis remains one of the most frequent and deleterious side effects in oncology patients undergoing chemotherapy. Patients who experienced intestinal mucositis underwent changes in their chemotherapy treatment, including dose reductions (45%), delays in therapy (71%), reduction in dose intensity (64%), and discontinuation of therapy (3%).[27,28] Recently, several studies have evaluated the effect of probiotics on mucositis, however, the results are contradictory.[29,30] In the present study, we examined whether probiotic administrations can prevent the development of severe 5-FU induced mucositis.

### Weight Loss

In our study, all normal mice gained BW on days 1–2 after saline administration, including those treated with *Lcr35* and *LaBi*. As previously reported, 5-FU injection resulted in marked weight loss and severe intestinal injury 5 days post-mucositis induction.[17] However, in those mice in the probiotics group, their BW loss’s intensity were significantly less than those in the 5-FU and saline groups. Our results were comparable to those published in the literature.[2,17]

### Diarrhea score

Walder et al reported that approximately one third of the oncology patients undergoing chemotherapy experienced severe diarrhea.[31] Regimens containing 5-FU has been documented with a higher risk for chemotherapy-induced diarrhea.[32] In our experiment, no diarrhea was noted in the 3 saline groups (with and without probiotics adminstrations). However, marked diarrhea developed in the three 5-FU groups 48 hours later. Diarrhea was attenuated and the diarrhea scores improved significantly after oral *Lcr35* and *LaBi* administrations.

### Cytokines analyses and mRNA expression

The exact pathogenesis of mucositis remain unclear. Some studies believed that it involved a five-stage process, including an initiation phase, a message generation phase, a signaling and amplification phase, an ulceration phase, and a healing phase.[27,33] Different cytokines are responsible for the various stages. Soares et al suggested two principles of mucositis development including the generation of reactive oxygen species which directly damage cells, tissue and blood vessels and the up-regulation of pro-inflammatory cytokines including TNF-α, IFN-γ, IL-1β and IL-6 causing further mucosal injury eliciting further tissue damage.[34] Proinflammatory cytokines such as IL-1β and TNF-α were also shown to play a role in amplifying the severity of chemotherapy-induced intestinal mucositis.[35] In our study, we demonstrated that those mice in 5-FU+saline groups had significantly higher levels of circulating pro-inflammatory cytokines and which decreased significantly after probiotics administration. It seems that both probiotic regimens attenuated the mucosal injury induced by 5-FU.

Furthermore, we found similar results when we determined the effect of probiotics treatment on the TNF-α, IL-6 and IFN-γ mRNA expressions in jejunum and colon tissues derived from 5-FU treated mice. Probiotics appear to attenuate the severity of intestinal mucositis induced in mice by 5-FU treatment through the inhibition of proinflammatory cytokines expression involved in the pathogenesis of mucositis.

### Histological Analysis: Villus height, crypt depth and goblet cells measurements

We showed that treatment with 5-FU caused significant villus shortening in our mice model. However, a repairing of damage in jejunal villi was observed following 5-FU treatment with probiotics (*Lcr35* or *LaBi*) administration. Interestingly, 5-FU-injected mice treated with *LaBi* caused a stronger effect when compared to *Lcr35* treatment. Besides, 5-FU significantly lengthened crypt of the intestine compared with the saline controls. With both *Lcr35* and *LaBi* treatments in 5-FU mice, the crypt depths were restored to the levels seen in those normal saline controls.

Besides villus shortening, treatment with 5-FU causes significant decreases the villus/crypt ratio in our mice model, which was comparable to the results in previous studies.[34] 5-FU markedly decreased the ratio in jejunal sections compared to the saline controls. These effects were alleviated by *Lcr35* and *LaB*i administrations in 5-FU-injected mice, although the levels did not reach to that in the normal saline groups.

Effects of chemotherapy-induced mucositis on villus height and crept depths varies and inconsistent in the literatures. Tazuke et al reported that jejunal crypts in healthy rats were markedly deepened by probiotics administration compared to normal controls.[36] However we found no difference in our study. Other studies demonstrated that both villus and crypt were lengthened as a result of increasing cell proliferation and villus elongation.[37] Tazuke et al also demonstrated an increase in small intestinal crypt cell proliferation following glutamine administration in a rat model of chemotherapy-induced mucosal injury.[36] These discrepancies might be due to differences in the administration routes, probiotic strains or regimens.

Effects of probiotics in the chemotherapy-induced colonic mucositis were not assessed histologically in our study. However, other studies in the literature demonstrated promising results. Bellavia et al analyzed the effects of supplementation with a mixture of *Lactobacillus casei* and *Bifidobacterium lactis* on the colon and liver of mice exposed to 2,4,6-trinitrobenzenesulfonic acid (TNBS) as an inflammatory agent. They demonstrated that exposure to TNBS obviously induced severe damage both in the colonic wall and liver parenchyma. However, probiotics supplementation significantly ameliorated the inflammation in the colonic mucosa.[10]

5-FU has been shown to negatively impact on mucin dynamics and might impede intestinal barrier function.[18] The main role of goblet cells is to secrete mucus in order to protect the mucous membrane.[38] Once secreted, mucins hydrate and gel in the lumen and generate a protective mucous barrier overlying the epithelial surface; this barrier protects the epithelium from mechanical and chemical stresses and allows transport between the luminal contents and the epithelium. A recent study of mucins and goblet cells in colitis suggests that they may be regulated by interactions between specific bacterial peptides and the gastrointestinal mucosa.[39] This result suggested a strong link between intestinal flora and secretion of mucin, which have both been shown to be affected in chemotherapy-induced mucositis. Stringer et al demonstrated a marked decrease in goblet cell number following 5-FU administration. Their study suggested the protective capabilities of the mucosal barrier might have been diminished following the depletion of stored mucins and probiotic-based therapies might be able to counter these deleterious effects.[18] In our study, we also demonstrated a marked decrease in goblet cell number in mice with 5-FU-induced mucositis, However, these effects were alleviated significantly following *Lcr35* and *LaBi* administrations, though the levels did not reach to that in the normal saline groups.

### Safety and translocation

Up-to-date probiotics are considered as harmless bacteria, potentially serious side-effects of probiotic therapies are possible, including development of sepsis, initiation of an extreme inflammatory response, growth of foreign bacterial colonies, presence of virulence factors within strains of probiotic bacteria, translocation of live bacteria into local tissues and the transfer of resistance genes throughout bacterial populations.[40–42] In the present study, no bacterial translocation was found in samples of blood, liver and spleen tissues. It seems the risk of systemic infection with probiotics administration in this mice model was not likely.

### Mechanisms

The exact mechanisms by which probiotics exert their beneficial effects remain unknown. The mechanisms may include prevention of pathogenic colonization in the gastrointestinal tract through competition for adhesion sites, re-establishing intestinal microflora after chemotherapeutic damage and the release of antimicrobial compounds.[11,43] Probiotics preserved the intestinal barrier in mouse models of colitis and reduced intestinal permeability in human patients with Crohn’s disease.[10,44] Keefe et al demonstrated that mucositis involved the loosening of tight junctions in the epithelial wall, and the subsequent loss of barrier function, facilitating the transfer of harmful luminal antigens into the surrounding intestinal tissue.[45]

Our previous research successfully demonstrated *Lactobacillus* were associated with the maintenance of the tight junction integrity and appearance.[14,15] Addition of *Lactobacillus* was able to reduce the LPS-induced inhibition of transepithelial electrical resistance (TEER) and reverse the change in tight junction protein ZO-1 expression. Ewaschuk and colleagues suggested that the *Bifidobacterium infantis* strain increased TEER, ZO-1 and occludin expression in normal T84 cells.[46] In this study we found TNF-α, IL-6 and IFN-γ mRNA expressions were up-regulated in tissue from 5-FU treated mice with mucositis. Probiotics appear to ameliorate the intestinal mucositis severity by inhibition the expressions of proinflammatory cytokines.

In the current study, we employed two probiotic formulations with concentrations of 1×10^7^ cfu/ml. These probiotics strains have been chosen because these strains are associated with the maintenance of the tight junction integrity.[14,15] Furthermore, they are widely used clinically in chronic gastrointestinal disorders with promising results. Since different probiotics demonstrate various multiple beneficial effects, it seems a single strain of probiotics will not be sufficient clinically. The microbial composition of the host may also affect probiotic efficacy. In a recent review, Prisciandaro et al proposed the promising idea that a combination of several probiotic strains may be most reliable and efficacious.[29]

Not all the research studies demonstrated beneficial effects of probiotics on chemotherapy-induced mucositis. Maioli et al demonstrated that *S*. *boulardii* was not able to prevent the effects of experimental mucositis induced by 5-FU.[5] Mauger et al and others also did not find beneficial effects using different species of probiotics in mucositis induced by 5-FU.[47,48] The differences could be explained by the use of different antineoplastic agents for inducing mucositis.

In this mouse model, our results showed a very clear and convincing protective effect and safety of probiotics on the chemotherapy induced mucositis. Previous studies in the literature seldom utilized the combination of probiotics and determined the effect of probiotics treatment on the expressions of pro-inflammatory cytokines in jejunal tissues derived from 5-FU treated mice. Furthermore, the safety of probiotics administrations were rarely emphasized and studied. To the best of our knowledge, this is the first study that investigated both the effectiveness and safety of probiotics in the treatment of chemotherapy-induced mucositis in a mouse model.

There are several limitations in our study. One limitation is that individual strains were not assessed to determine their possible contributions for the observed effects. Another limitation is that we focused on the histological effects of probiotics on small intestines; other parts of the gastrointestinal tract such as colon and stomach specimens were not examined. Also, this study did not address the possible mechanisms by which probiotics exert their beneficial outcomes such as the effects on tight junction proteins and TEER. These areas should be investigated in future experiments.

Further studies should focus on identification of the most suitable probiotic strains and determining the importance of strain specificity and dosage. In addition, exploration of the probiotics effects on tight junction expression and intestinal permeability should be conducted to better elucidate the underlying mechanisms. More clinical works are needed to demonstrate the beneficial effects of different probiotics and elucidate the correct dosing regimens for the management of chemotherapy-induced mucositis.

## Conclusions

Our results show that oral administration of probiotics *Lcr35* and *LaB*i can ameliorate chemotherapy-induced intestinal mucositis in a mouse model. This suggests probiotics may serve as an alternative therapeutic strategy for the prevention or management of chemotherapy-induced mucositis in the future.
