# Correction: Amelioration of Chemotherapy-Induced Intestinal Mucositis by Orally Administered Probiotics in a Mouse Model

**DOI:** 10.1371/journal.pone.0141402

**Published:** 2015-10-20

**Authors:** Chun-Yan Yeung, Wai-Tao Chan, Chun-Bin Jiang, Mei-Lien Cheng, Chia-Yuan Liu, Szu-Wen Chang, Jen-Shiu Chiang Chiau, Hung-Chang Lee

The captions for Figs 5 and 6 are incorrectly switched. The caption for Fig 5 should be the caption for Fig 6, and the caption for Fig 6 should be the caption for Fig 5. Please see the corrected captions here.

**Fig 5. A: Representative histological sections of jejunum showing the goblet cells with haematoxylin and eosin stain in mice on day 5 challenged with 5-FU (IP)**. They were fed with probiotics (Lcr35orLaBi) or saline. The arrows indicated goblet cells. The image acquisition phase was done with a 20x magnification objective. Scale bar = 50μm. B: Jejunal goblet cells after staining were counted. Values were represented as mean±SEM and were analyzed using one-way ANOVA.

**Fig 6. Up-regulations of IL-6, IL-1βand TNF-αin mucositis mice were followed after injection with 5-FU**. Mucositis mice were fed with (+) or without (−) probiotics. Gene expressions of IL-6, IL-1βand TNF-αwere determined by Q-PCR (A) jejunum tissue (B) colon tissue. Induction of cytokine expressions were presented as RQ compared to 18sRNA housekeeping gene expression. The data with different superscripted letters are significantly different based on the one-way ANOVA.
